# Intercultural sensitivity in Chilean healthcare profession students

**DOI:** 10.1186/s12909-024-05443-w

**Published:** 2024-04-26

**Authors:** Patricia Pineda, Maura Klenner, Gerardo Espinoza, Rodrigo Mariño, Carlos Zaror

**Affiliations:** 1 Faculty of Dentistry, Department of Pediatric Dentistry and Orthodontics, Universidad de La Frontera, Temuco, Chile; 2 Center for Research in Epidemiology, Economics and Oral Public Health (CIEESPO), Temuco, Chile; 3 Departamento de Lenguas, Universidad de La Frontera, Literatura y Comunicación, Temuco, Chile; 4 Faculty of Medicine, Department of Public Health, Universidad de La Frontera, Temuco, Chile; 5 University of Melbourne, Melbourne Dental School, Melbourne, Australia; 6 University of Puthisatra, Phnom Penh, Cambodia

**Keywords:** Intercultural sensitivity, Health profession students, Curricula, Chile

## Abstract

Each culture has unique health care related values, habits, perceptions, expectations, norms, etc., that makes cultural competence an important attribute to be developed by healthcare professionals, to ensure they provide effective treatment. Intercultural sensitivity (IS) is the affective dimension of cultural competence. The objective of this study is to explore the self-perceived level of IS in first and last year students of three health sciences professions (i.e., Dentistry, Medicine, and Nursing) at the Universidad de la Frontera, Temuco, Chile. This study adopted a cross-sectional design and a group comparison (e.g., year of study). 312 students completed the Intercultural Sensitivity Scale (ISS). Findings showed that overall ISS scores ranged from 1.83 to 4.94, with a mean score of 4.11 (s.d. 0.43). Group comparison between first and final year students showed statistically significant differences (4.18 vs. 4.00; *p* < 0.001). Medical and nursing students had a significantly higher overall mean IS score compared to dental students (4.21 and 4.16, respectively vs. 4.02; *p* < 0.01). There were also significant differences between three factors (interaction engagement; interaction confidence; and interaction enjoyment) by healthcare profession. These findings allow for discussion of the need for explicit incorporation and development of cultural competence in on health care professional curricula. Longitudinal research is needed to explore how IS changes over time, along with generating qualitative data from the student populations IS experiences and exposure.

**Supplementary Information:**

The online version contains supplementary material available at 10.1186/s12909-024-05443-w.

## Background

Within the current trend of globalization and greater population mobility (i.e., migrations) both within and between countries, the importance of cultural diversity constitutes a challenge that healthcare professionals and teams must address as part of their everyday healthcare activities. Different approaches may be required to address healthcare problems of culturally diverse groups compared to mainstream groups [1, 2]. Each culture has unique values, habits, perceptions, expectations, norms, etc. about health care, which makes cultural competence of great importance for healthcare professionals [3]. Although cultural competence is developed in a process, its acquisition must begin during the education of the healthcare professional [4, 5].

In the field of healthcare, Cultural Competence (CC) is defined as “a process in which the health care provider continually strives to achieve the ability to work effectively within a patient’s cultural context“ [6]. Another definition describes CC as the ability to work collaboratively with people who have different cultures and points out that intercultural competence is a construct, which has cognitive, affective and behavioral elements to understand, appreciate, and accept cultural diversity [7]. Intercultural sensitivity (IS), the affective dimension of intercultural competence, has been described as its main dimension [7].

Chen and Starosta conceptualized intercultural sensitivity as the “capacity of a person to develop a positive emotion towards the understanding and appreciation of cultural differences that promote appropriate and effective behaviors in intercultural communication” [8]. People with high levels of IS can provide holistic care to the people they have to care for, have developed elevated levels of moral and ethical sensitivity and complex empathy skills, and are conscious of their own professional responsibilities. An individual who respects cultural diversity has low levels of ethnocentrism,^1^ and as a consequence, shows increased levels of IS [9].

IS denotes both visibility and affirmation of cultural differences, as well as the rejection of ethnocentric perspectives [10]. IS contributes to the development of abilities to identify the importance of recognizing, comprehending, and appreciating others in their differences, putting oneself in somebody else’s place in other to in order to access different worldviews [11].

In any modern society, there are four articulated phenomena that determine the need to teach and display intercultural skills in healthcare: (i) migration; (ii) the different ethnic groups; (iii) cultural diversity; and (iv) the biomedical model inserted in the Western health system [1]. Furthermore, this scenario of multiculturalism and diversity is also emphasised within each community in terms of rural or urban areas, social, economic, ethnic, and religious differences, among others, which can be challenging for many healthcare professionals. On the one hand, it may be difficult for them to understand the beliefs and healthcare practices of the people they care for and, on the other, they may be concerned about the lack of success in adherence to treatments [12].

As part of a larger study to help unravel the cultural issues faced by healthcare profession students when treating culturally diverse patients [13], this study aims to explore the self-perceived level of IS in first and last year students of three health sciences courses (Dentistry, Medicine, and Nursing) at the Universidad de la Frontera (UFRO), Temuco, Chile. The study will establish a baseline for future comparisons. Additionally, the study will explore how cultural competency is influenced by the curriculum as they progress through their health training, using the first and last year as an approximation (proxy) of the length of exposure. These perceptions of IS, together with the review of the cultural challenges faced in implementing healthcare curricula in the context of a diverse patient base [13], may inform future curricular reviews to fulfil healthcare students’ needs. Furthermore, latest data indicates that 12.8% of the Chilean population declared some First nation identity [14]. This cultural diversity is increased especially in regions with a high percentage of the population belonging to First nation people such as La Araucanía Region, which stand out for the high percentage of the population that self-identify as belonging to a specific ethnic group (34.3%) [14].

## Methods

This study adopted a cross-sectional design and a group comparison (e.g., year of study) to study the IS of Medical, Nursing, and Dental students. Following ethics approval from the UFRO Research Ethics Committee (Ref Nº: 072/19). The study surveyed all first and final year healthcare professions (i.e., Medical, Nursing, and Dental), 18-year-old or older students studying at UFRO, using anonymous self-completed questionnaires, including the Intercultural Sensitivity Scale (ISS). These cohorts’ selection allows an approximation (proxy) of length of exposure to explore the development of students’ IS over time. Data were collected between June 2021 and April 2022.

The Intercultural Sensitivity Scale (ISS) is a self-report scale that has been extensively used in different areas to assess the development of IS [8]. ISS assesses intercultural sensitivity as the affective dimension of intercultural competence. The theoretical structure of this scale was based on five dimensions, namely: interaction engagement; respect for cultural differences; interaction confidence; interaction enjoyment; and interaction attentiveness, as shown in Appendix 1. ISS is a 24-item scale organised in a 5-point Likert-type scale (1 = strongly disagree; 2 = disagree; 3 = neutral; 4 = agree; 5 = strongly agree). Some items are coded reversely in the scale. Negative questions were reversed to calculate the ISS score. Scale scores range from ‘1’, the lowest to ‘5’, the highest. There is not a cut-off value of the scale. A high score indicates higher intercultural sensitivity [15]. The ISS was linguistically adapted and validated to be used in the Chilean context [11].

Due to the COVID 19 restrictions, the instrument was redesigned to be completed online. All first and final year students were briefed about the objectives of the study and invited to participate. Participants were requested to complete the online questionnaire anonymously, using a self-assigned code. Data collection was done via the QuestionPRo platform [15].

Apart from the variables of age, sex, year of study (first and last of each course), and course (medicine, dentistry, or nursing), region of residence (out of the 16 Regions in which Chile is divided administratively, categorised as **‘**Araucania’; ‘Los Lagos’; ‘Bio-Bio’; and ‘Other’). Students’ ethnicity was also collected (Broad Chilean; Mapuche; and Other). Participants’ family group income was determined using seven monthly income levels in Chilean pesos (‘$300,000 or less’; ‘$300,001 to $600,000’; ‘$600,001 to $1,000,000’; ‘$1,000,001 to $1,500,000’; ‘$1,500,001 to $2,000,000’;’$2,000,001 to $3,000,000’; and ‘More than $3,000,000’). Participants were also asked about the type of secondary education. Secondary education in Chile has three types funding: ‘Private’; ‘Publicly subsidized private’; and ‘Municipal’. Municipal funds focus on lower socio-economic status (SES) individuals, while private education generally covers those in higher SES groups [16].

The dependent variable represented by the overall IS score and eight socio-demographic and study variables were included in the analysis. Five intercultural sensitivity scores were computed by calculating average responses across the five intercultural sensitivity dimensions. Additionally, an overall intercultural sensitivity score was computed by calculating average responses across all the five intercultural sensitivity factors.

The statistical analysis describes the distribution of the socio-demographic and study variables. To examine whether any independent variables (e.g., year of study) had the same pattern of ISS mean, Analysis of Variance (ANOVA) (continuous measures) were employed. A significant ANOVA was followed by post-hoc comparisons using Tukey’s Honestly Significant Differences (HSD) tests. To explore associations between nominal and ordinal variables (e.g., sex and year of education), chi square analysis was applied. To test if any combination of the various socio- demographic, and study variables, provided a multivariate explanation of the IS score, a linear regression model was fitted using a stepwise selection method. Variables included in the regression model were based on a combination of factors, including the theoretical framework the study, and the literature. However, variables in the final model were retained on statistical criteria. A probability value of 0.05 or smaller was considered to be statistically significant. Data manipulation and analyses were conducted using SPSS PC (Version 27.0).

## Results

Four hundred and nineteen students were invited to participate in the survey, with 210 in the first year and 209 in the last year of their course. The overall response rates were 93.8% and 55%, respectively. Among those who completed the survey, 312 cases were included in the analysis after excluding six incomplete forms. Of those, there were 105 nursing students: 76 (response rate: 100%) were in the first year and 29 in the final (response rate: 49.2%) year; and 143 dental students: 68 in first year (response rate: 97.1%) and 75 in the final year (response rate: 100%). There were also 64 medical students: 53 in the first year (response rate: 74.6%) and 11 students in the final year of their studies (response rate: 13.8%).

The mean age of participants was 23.1 (s.d. 3.3) years, and most of them were females, accounting for 68.7%. Publicly subsidized private schooling was the most common type of secondary education among students (54.8%), followed by public schooling (33.0%). The remaining 12.2% attend non-subsidized private schools. Most participants were from the La Araucania Region (77.5%), followed by the Los Lagos Region (7.4%), and Bio-Bio Region (6.4%). The remaining participants were from other 10 Regions (8.7%). With regard to ethnic background, the majority self-identified as without any cultural group different to broad Chilean (78.3%), and 20.7% (*n* = 65) self-identified with another culture. The majority of these (86.3%) as Mapuche people and 7.5% as other cultures. The remaining 6.2% did not specify their ethnicity. Table 1 presents the distribution of students by socio-demographic and study characteristics.

**Table 1:** Distribution of the sample by career and year o study according to socio-demographic variables

	Nursing		Dentistry		Medicine		Total
Year of study	First (*n* = 76)	Final (*n* = 29)	First (*n* = 68)	Final (*n* = 75)	First (*n* = 53)	Final (*n* = 11)	*N* = 312
Mean age (years)	20.4 (1.3)	24.6 (1.5)	21.1 (1.3)	27.6 (1.7)	21.7 (2.3)	26.8 (2.1)	23.1(3.3)
Sex (%)							
Female	78.7	93.1	58.8	63.5	60.4	72.7	68.7
Cultural background (%)							
Mapuche (Yes)	36.8	13.8	17.6	10.7	9.4	0.0	18.2
Income Chile $ (%)							
300,000 or less 300,001–600,000 600,001–1,000,000 1,000,001–1,500,000 1,500,001–2,000,000 2,000,001–3,000,000 More than 3,000,000	36.8 22.4 11.8 15.8 6.6 2.6 3.9	10.3 55.2 20.7 6.9 0.0 6.9 0.0	33.8 35.4 6.2 6.2 10.8 1.5 6.2	12.5 22.2 15.3 18.1 13.9 9.7 8.3	5.7 18.9 24.5 15.1 5.7 15.1 15.1	0.0 9.1 9.1 9.2 0.0 45.5 27.3	21.2 27.2 14.4 13.1 8.2 8.2 7.8
Type of education (%)							
Private Publicly subsidized Municipal	3.9 46.1 50.0	6.9 55.2 37.9	14.7 45.6 49.7	10.7 65.3 24.0	20.8 62.2 17.0	36.4 63.6 0.0	12.2 54.8 33.0
Region of residence (%)							
Araucania Los Lagos Bio-Bio Other	90.8 5.3 0.0 3.9	86.2 6.9 0.0 6.9	68.7 10.4 11.9 9.0	70.7 6.7 13.3 9.3	69.8 9.4 3.8 17.0	100.0 0.0 0.0 0.0	77.5 7.4 6.4 8.7

The overall ISS scores ranged from 1.83 to 4.94, with a mean score of 4.11 (s.d. 0.43). The mean scores by subscales were as follows; 4.17 (s.d. 0.53) in the interaction engagement subscale; 4.52 (s.d. 0.51) in the respect for cultural differences subscale; 3.66 (s.d. 0.69) in the subscale of interaction confidence; 4.44 (s.d. 0.58); in the interaction enjoyment subscale, and 3.78 (0.65) in the interaction attentiveness subscale (Table 2). Overall, there were statistically significantly differences when comparing first and final year’s ISS scores (4.18 vs. 4.00; *p* < 0.001). Differences by healthcare profession were also evident. Medical and nursing students had a significantly higher overall mean ISS score compared to dental students (4.21 and 4.16, respectively vs. 4.02; *p* < 0.01). No significant associations were found in the ISS by ethnic group, by Region, by sex, or by type of secondary education.

**Table 2:** Mean Intercultural Sensitivity Scale (ISS) score by dimensions, health profession, and year of study (s.d.)

Dimensions¥	Nursing		Dentistry		Medicine		Total by factor
Year of study	First	Final	First	Final	First	Final	
Interaction engagement	4.26 (0.48)	4.16 (0.49)	4.08 (0.58)	4.05 (0.57)	4.35 (0.47)	3.96 (0.43)*	4.17 (0.53)**
Respect of cultural difference	4.60 (0.47)	4.37 (0.42)*	4.52 (0.51)	4.41 (0.57)	4.73 (0.35)	4.21 (0.56)***	4.52 (0.51)
Interaction confidence	3.76 (0.73)	3.61 (0.60)	3.64 (0.62)	3.48 (0.72)	3.84 (0.67)	3.52 (0.51)	3.66 (0.69)*
Interaction enjoyment	4.57 (0.48)	4.24 (0.46)**	4.42 (0.59)	4.29 (0.68)	4.66 (0.50)	4.12 (0.69)**	4.44 (0.58)*
Interaction attentiveness	3.81 (0.63)	3.92 (0.66)	3.75 (0.69)	3.73 (0.66)	3.83 (0.62)	3.49 (0.69)	3.78 (0.65)
**Total by year of study**	4.20 (0.40)	4.06 (0.41)	4.06 (0.45)	4.00 (0.47)	4.29 (0.34)	3.86 (0.43)***	
**Overall ISS score by career**	4.16 (0.40)		4.03 (0.46)		4.21 (0.39		4.11 (0.43)**

¥: ANOVA; *: *p* < 0.05; **: *p* < 0.01; ***: *p* < 0.001

By ISS dimension, except for dental students, within each profession there were significant differences between the first and final year (See Table 2). Furthermore, there were also significant differences between three factors (i.e., interaction engagement; interaction confidence; and interaction enjoyment) by course. However, only medical students reached significance levels with the overall score by year (4.29 vs. 3.86 *p* < 0.001).

There were also statistically different ISS scores between the first-year student cohorts (i.e., nursing compared with dental). First year nursing and medical students scored higher than dental students (4.20 and 4.29, respectively vs. 4.06; *p* < 0.05). On the other hand, when scores from final year students were compared, there were no significant differences (Table 2).

To better understand the variance in the overall ISS score, eight socio-demographic and course variables (age, sex, income, type of education, region of residence, profession, cultural background, and year of study), were entered into a multiple linear regression model. However, age was dropped from the model because of high collinearity with the year of study variable. The final model had two significant variables (i.e., course, and year of study) [F(2,294) = 8.411 *p* < 0.001]. The resulting model indicated that, after controlling for other independent variables in the model, those who had the highest IS score were first year students not studying dentistry. The variance accounted for, using the full model, was 5.4% (adjusted R^2^ = 0.054) (See Table 3).

**Table 3:** Final multivariate model identifying the Intercultural Sensitivity Scale score

Independent variable	Multiple regression coefficient B (Std. Error)	*p*-value
First year student (No = 0; Yes = 1) Dental student (No = 0; Yes = 1) Intercept	0.146 (0.05) -0.108 (0.05) 4.069 (0. 05)	0.006 0.034 0.0001

Adjusted r2 = 0.051

## Discussion

A fundamental element to achieve Intercultural competence is the development of intercultural sensitivity [7]. This is the first Chilean study to explore the level of IS which may be required to safely handle culturally diverse health situations. This study reports on the measure of healthcare students’ level of IS. Present findings would indicate that, across all healthcare professions, students tended to exhibit lower levels of self-reported IS in the final year of their studies compared to the first year. It has been reported that explicit instruction on IS in curricula is required to prompt this aspect of CC in healthcare [17]. As it was reported in other manuscript pertaining to this research [13], it was found that the sample reported on this study had been exposed to a more traditional biomedical approach in their professional formative process, which did not include the development of CC in a consistent and explicit manner across the curricula of the three programs but is developed in a more tangential mode. Alternative explanations for the decreasing IS levels may be that first-year students had been more exposed to intercultural encounters during their secondary education before entering university around 2020, due to the increasing immigration waves into the country prior to the COVID-19 pandemic [18].

Intercultural competence can be developed early through schooling processes [19] In Australia, newly graduated dental practitioners, when surveyed about their training in cultural competence, said that the current curricula did not focus enough on cultural issues and that additional training would be of benefit [20]. Furthermore, some of them indicated that most of their cultural and social perceptions evolved from their upbringing, high school and primary school education, and past personal experiences, rather than from exposure to the dental curriculum [20]. This introduces the influence of socio-demographics, personalities, experiences, and other factors. On the other hand, final year students may have had fewer formative opportunities to develop intercultural competencies due to the COVID-19 pandemic and lockdowns starting at the time they were about to start immersing their placements, internships, and clinical work. This, in turn, may have affected their IS.

In the present study, dental students reported lower IS scores compared to nursing and medical counterparts. This is consistent with previous publications from this study, which reports that the dentistry curriculum showed the majority of CC related themes are treated mainly at emerging and intermediate depths of coverage [13].

This study also revealed that students from La Araucanía, where this university is located, and students who report being Mapuche, do not obtain higher levels of IS. High levels of IS have been linked to multicultural societies in which cultural diversity is socially and institutionally accepted and appreciated [21]. Although La Araucanía Region has the country’s largest proportion of First Nations people, it has been recognized as the center of historical struggles between the Chilean State and First Nations people, particularly Mapuche people, for the recognition and incorporation of an intercultural view in national policies [22]. This situation might affect the general population’s IS levels, since the concept of interculturality could be related to social discontent. However, the majority of the students were from contiguous regions (i.e., Los Lagos, Bio-Bio). Therefore, this hypothesis may require further exploration.

The lack of significant difference in IS scores between those students who self-reported Mapuche ancestry and those who did not, questions the concept of ethnic and cultural equivalence of higher education students and may suggest that before or during the process of professionalization there is a common cultural profile to which the majority of students subscribed [4]. This calls for the examination of a critical question: Are healthcare profession students embedded in a cultural system, defined by their ancestries, or are there universal characteristics that are unique to healthcare profession students? This remains an unanswered empirical question which would need to be answered with further studies. However, it is also possible that the IS instrument loses cultural equivalence when applied to different ethnocultural populations [23].

Although the study reached an overall response rate higher than expected (i.e., 60%) [24], limitations of the study include low response rates, particularly, the final year medical students’ responses, relative to the size of the final year medical student population, and to a lesser extent the low response rate for last year nursing students. The invitation to participate was sent during the COVID-19 pandemic lockdown in Chile, which may have contributed to low response rates. On the other hand, low response rates are not unexpected in online surveys. Response rates to online surveys about oral health are within the range of 2.5–26% [25, 26].

Another limitation was the self-reported nature of the responses, which may not be an accurate reflection of the actual students’ IS [27, 28]. Furthermore, the cross-sectional nature of this study precludes a strong conclusion about IS exposure to healthcare professional education, or the use of year of study as a proxy for years of exposure to opportunities to develop sensitization to issues of cultural diversity during professional training. Another concern is that participants were students at one university (i.e., UFRO) only. As a result, conclusions drawn from this study may not be representative of the IS of all Chilean healthcare profession students [29].

Thus, it is not implied that a final definitive model of IS among Chilean healthcare profession students has been developed, rather his study raises some factors to be investigated in the future. Nonetheless, despite its limitations, we believe that the current approach was adequate given the exploratory nature of the study. The primary goal of this study was to describe the perceptions of IS among healthcare professions students. The present results show consistent findings with a qualitative review of the healthcare professions curricula at UFRO [13]. Together, these efforts provide robust evidence about the need to upsurge formative experiences to increase students’ awareness and experience to provide a culturally safe encounter when treating a patient from a culture different to the student’s own.

Research involving the collection of longitudinal data is needed to explore how IS changes over time. Future studies should also involve qualitative data collection from student populations’ IS experiences and exposure. This analysis would generate opportunities for a broader understanding of their intercultural sensitivity, experience, and other contextual issues. Additionally, cooperation with other dental, medical, and nursing schools/Faculties in Chile or overseas would be beneficial in confirming the present results and understanding the influence of IS education on students as the future health workforce.

Against a background of increased international mobility and recognition of indigenous nations within the territory, there is a growing interest in Chile in reviewing the extent to which cultural competence is covered in the healthcare professions curricula to meet the standards and expectations of accreditation organizations [30, 31]. While this approach is still incipient, from this perspective, this research is significant because it is among the few to comparatively examine and document IS among healthcare profession students in Chile. This information is central to developing educational programs for the future health workforce. It is also expected that the findings of this project will assist in the development of accreditation standards, policies, and professional competencies for healthcare professionals.

### Electronic supplementary material

Below is the link to the electronic supplementary material.

Supplementary Material 1

## Data Availability

Ethics approval was granted on the basis that only researchers involved in the study and could access the de-identified data. The minimum retention period is 5 years from publication. Supporting documents are available upon request to the corresponding author.

## Footnotes

1 Ethnocentrism involves a tendency to evaluate and judge other cultures based on the standards and values of one’s own culture, resulting in a biased interpretation and understanding of other cultures. Merriam-Webster.com Dictionary, Merriam-Webster, https://www.merriam-webster.com/dictionary/ethnocentric. Accessed 20 Mar. 2024.

## Abbreviations

ANOVA: One-way analysis of variance
SES: Socio-economic status
UFRO: Universidad de La Frontera
