# Supplementary material for: Intercultural sensitivity in Chilean healthcare profession students
Source: BMC Med Educ. 2024 Apr 26;24:467. doi: 10.1186/s12909-024-05443-w (PMC11055216; doi:10.1186/s12909-024-05443-w)
Supplement: Supplementary file 1 — Supplementary Material 1 [file 12909_2024_5443_MOESM1_ESM.docx]

Appendix 1. Intercultural sensitivity Scale (ISS).

**Factor 1. Intercultural engagement**

Item 1. I enjoy interacting with people from different cultures.
Item 11. I tend to wait before forming an impression of culturally-distinct counterparts.
Item 13. I am open-minded to people from different cultures.
Item 21. I often give positive responses to my culturally-different counterpart during our interaction.
Item 22. I avoid those situations where I will have to deal with culturally-distinct persons.
Item 23. I often show my culturally-distinct counterpart my understanding through verbal or nonverbal cues.
Item 24. I have a feeling of enjoyment toward differences between my culturally-distinct counterpart and me.

**Factor 2. Respect of cultural difference**

Item 2. I think people from other cultures are narrow-minded.
Item 7. I do not like to be with people from different cultures.
Item 8. I respect the values of people from different cultures.
Item 16. I respect the ways people from different cultures behave.
Item 18. I would not accept the opinions of people from different cultures.
Item 20. I think my culture is better than other cultures.

**Factor 3. Interaction confidence**

Item 3. I am pretty sure of myself in interacting with people from different cultures.
Item 4. I find it very hard to talk in front of people from different cultures.
Item 5. I always know what to say when interacting with people from different cultures.
Item 6. I can be as sociable as I want to be when interacting with people from different cultures.
Item 10. I feel confident when interacting with people from different cultures.

**Factor 4. Interaction enjoyment**

Item 9. I get upset easily when interacting with people from different cultures.
Item 12. I often get discouraged when I am with people from different cultures.
Item 15. I often feel useless when interacting with people from different cultures.

**Factor 5. Interaction attentiveness**

Item 14. I am very observant when interacting with people from different cultures.
Item 17. I try to obtain as much information as I can when interacting with people from different cultures.
Item 19. I am sensitive to my culturally distinct counterpart’s subtle meanings during our interaction.
